# Health Qigong Mawangdui Guidance can improve pelvic floor muscle function and quality of life in females with stress urinary incontinence: A randomized controlled trial pilot study

**DOI:** 10.1097/MD.0000000000037671

**Published:** 2024-04-12

**Authors:** Ming Xu, Xu Zhang, Yue Zhuo, Wanrong Wu, Qiong Liu, Shuang Qin, Zhuan Long, Kun Ai, Ya Li, Hong Zhang

**Affiliations:** a College of Acupuncture-Moxibustion and Rehabilitation, Hunan University of Chinese Medicine, Changsha, China; b The First Hospital of Hunan University of Chinese Medicine, Changsha, China; c College of Sports Art, Hunan University of Chinese Medicine, Changsha, China; d Changsha Central Hospital, Changsha, China.

**Keywords:** Chinese medicine, pelvic floor muscle function, Qigong, quality of life, stress urinary incontinence

## Abstract

**Background:**

Urinary incontinence (UI) is a great problem of public health, especially for women’s quality of life. UI afflicts at least 21.6% of the global population, and more than half of the UI is related to female stress urinary incontinence (SUI). Mawangdui Guidance plays an important role in preventing diseases and maintaining health.

**Methods:**

Sixty female patients with SUI were randomly divided into a control group (n = 30) and an experimental group (n = 30). Patients in both groups were treated with basic rehabilitation therapy under the guidance of rehabilitation therapists who were trained in Mawangdui Guidance, based on the former, the experimental group was taught to exercise Mawangdui Guidance(including selected movements: “Qishi,” “Longdeng,” “Chishi,” and “Yinyao”), while the control group performed Kegel exercise with a procedure of 20 min, six times per week for 6 weeks. The function was mainly evaluated by the 1 h pad-test, incontinence quality of life questionnaire (I-QOL), and international consultation on incontinence questionnaire urinary incontinence short form (ICI-Q-SF). In addition, evaluation of pelvic floor muscle function was also included in our assessment.

**Results:**

The leakage of urine in the 1 h pad-test was significantly decreased in both two groups after treatment (*P* < .05), and the urine leakage in the experimental group was significantly less than that in the control group (*P* < .05). The muscle strength of type I and II muscle fibers of the pelvic floor, intravaginal pressure, and I-QOL score in both two groups were increased after treatment; moreover, the experimental group was more significant than the control group (*P* <.05). The fatigue degree of type I and type II muscle fibers of the pelvic floor, and the ICI-Q-SF score in both groups were significantly improved after treatment (*P* < .05); however, there were no differences between these two groups. The total effective rate of the experimental group was 90.00%, and 76.67% in the control group (*P* <.05).

**Conclusion:**

Mawangdui Guidance can effectively improve the function of pelvic floor muscle, improve the ability of urine storage and control, and alleviate the symptoms of female patients with SUI. However, the international research on Mawangdui Guidance is very limited, and more in-depth research is needed.

## 1. Introduction

Urinary incontinence (UI) is a common health problem that has been paid more and more attention to all over the world. The International Continence Society defines stress urinary incontinence (SUI) as an involuntary and uncontrollable leakage of urine caused by a sudden increase in abdominal pressure due to sneezing, coughing, laughing, or exercise. Nowadays, it is believed that the pathogenesis of SUI mainly includes anatomical disorders caused by overactivity of the bladder neck and urethra or intrinsic sphincter deficiency of the inherent urethral.^[1]^ UI afflicts at least 21.6% of the global population, which more than half of the UI is related to female SUI.^[2,3]^ A full sampling survey of women over 30 years old in South Korea showed that the prevalence of UI was as high as 40.8%, which SUI accounts for about 22.9%.^[4]^ The results of the Japanese epidemiological survey in 2008 showed that the prevalence rate of SUI was about 19.3%,^[5]^ however, in China, the consultation rate of SUI patients within 5 years is only 8%.^[6]^

Current SUI treatments are divided into 2 categories that are not mutually exclusive: surgical and conservative treatment. National Institute for Health and Clinical Excellence suggested that patients with UI should be treated with conservative treatment first,^[7]^ such as pelvic floor muscle training, pelvic floor electrical stimulation, lifestyle intervention, drug therapy, and so on.^[8]^ However, long-term use of drugs may lead to toxic side effects. The intervention of behavior and lifestyle is safe but requires very high, often unattainable, patient compliance. As a result, the most widely used and easily accepted conservative treatment is pelvic floor tissue electrical stimulation or pelvic floor muscle active movement therapy. The treatment of SUI is a long-term process, and it is very important to guide patients to carry out the correct self-rehabilitation exercise. Nowadays, there is not much strategy or research on this problem, which is worthy of our more attention.

As the prototype of the traditional Chinese Qigong exercise routine, Mawangdui Guidance has a very long history, which can date back to the Qin and Han dynasties in China (221 BC–220 AD).^[9]^ Some research indicated that Mawangdui Guidance plays an important role in preventing diseases and maintaining health,^[10–12]^ even in preventing dementia in the old.^[13,14]^ The practice of Mawangdui Guidance includes 12 movements and it takes about 7 minutes to do full set of exercises, which is challengeable for patients to learn a full set of movements. In addition, it remains to be verified whether patients can adhere to complete set of movements. Mawangdui Guidance is an important part of traditional Chinese medicine, which plays an important role in strengthening the function of pelvic floor muscles. We selected 4 actions including “Qishi,” “Longdeng,” “Chishi,” and “Yinyao” that can make the pelvic floor muscle, low back muscle, and abdominal muscle contract independently, enhance or improve the coordination of muscle strength around the pelvic floor.

The main purpose of this study is to observe the clinical function of Mawangdui Guidance in the treatment of SUI in females, what’s more, to explore the mechanism of Mawangdui Guidance on SUI. Our study combines kinesiology with traditional Chinese Qigong and passive electrical stimulation with active exercise therapy. We hope to prove that biofeedback electrical stimulation of pelvic floor muscle combined with Mawangdui Guidance may be a new and promising comprehensive treatment. If so, it will become a new strategy for prevention and treatment of SUI.

## 2. Experimental methods

### 2.1. Participants

The study was registered in the Chinese Clinical Trial Registry (ChiCTR2000034768). This study was reviewed and received ethics clearance through the Ethics Committee of the First Hospital of Hunan University of Chinese Medicine (HN-LL-KY-2020-019-01).

**Figure 1.**
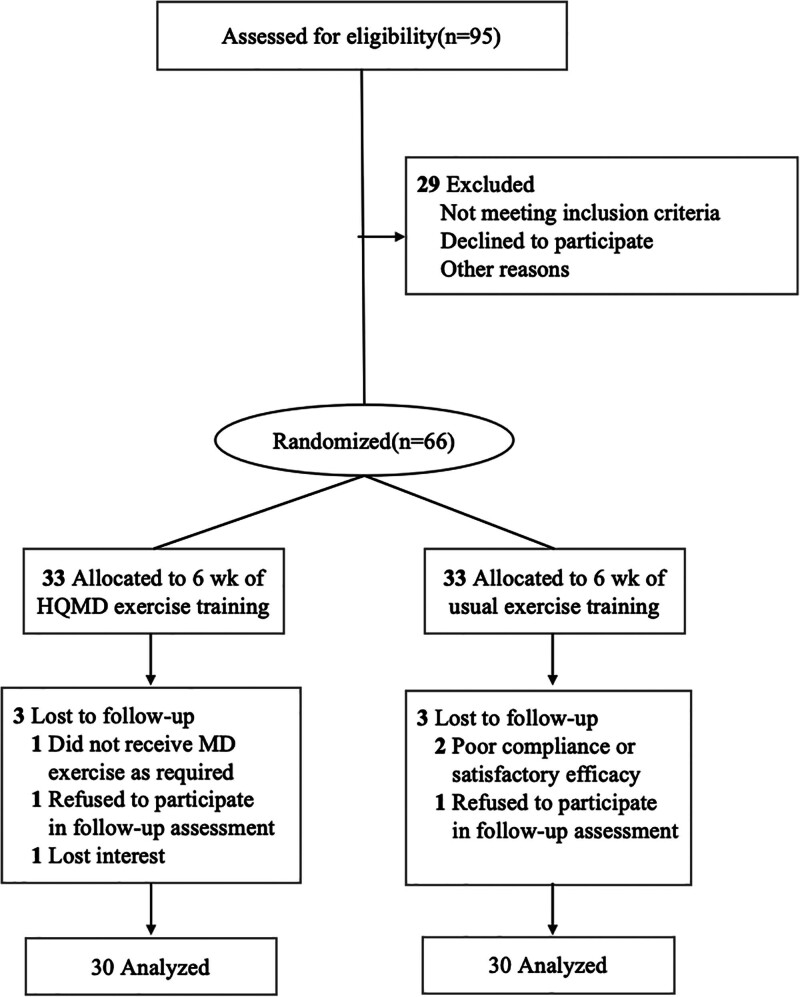
Flow diagram depicting the study design.

By excluding cases that did not meet the criteria, we eventually included 60 patients for the study. These 60 female patients suffering from SUI were recruited from the Department of Rehabilitation of First Affiliated Hospital of Hunan University of Chinese Medicine, including the outpatient clinic, inpatient department, and community hospital. Random concealed envelopes were prepared by using a random number table. Under the condition that patients and their family members signed the informed consent, patients were divided into the control group (n = 30) and the experimental group (n = 30) according to the order of being admitted to hospital. The specific exclusion and grouping methods are shown in Figure 1. The baseline characteristics of the participants are depicted in Table 1, including the age, course of the disease, body mass index, and the number of prior pregnancies, there were no statistically significant differences in the general conditions between the 2 groups (*P* > .05).

**Table 1:** Participant baseline characteristics.

Characteristic	Control group (n = 30)	Experimental group (n = 30)
Age, mean (SD), year	39.67 (6.77)	38.53 (8.20)
Body mass index, mean (SD), kg/m^2^	26.70 (2.94)	27.17 (3.36)
Course, mean (SD), month	7.20 (3.05)	7.37 (2.46)
The number (%) of births		
0	0	0
1	13	11
2	15	18
≥3	2	1
Amount of urine leakage measured by the 1-h pad-test, mean (SD), g	4.61 ± 2.67	4.52 ± 2.39
Fatigue degree of pelvic floor muscle fibers, mean (SD)		
Type I	–0.767 ± 10.7	–2.97 ± 9.41
Type II	–1.83 ± 12.80	–0.27 ± 9.78
Pelvic floor muscle strength, type I, II, n (%)		
0	4 (13.33%), 4 (13.33%)	4 (13.33%), 2 (6.67%)
I	7 (23.33%), 1 (3.33%)	12 (40.00%), 1 (3.33%)
II	3 (10.00%), 0 (0%)	3 (10.00%), 4 (13.33%)
III	2 (6.67%), 3 (10.00%)	3 (10.00%), 6 (20.00%)
IV	6 (20.00%), 10 (33.33%)	4 (13.33%), 11 (36.67%)
V	8 (26.67%), 12 (40.00%)	4 (13.33%), 6 (20.00%)
Vaginal dynamic pressure, mean (SD)	61.37 ± 20.69	57.93 ± 12.27
I-QOL, mean (SD)	34.83 ± 6.37	35.03 ± 4.97
ICI-Q-SF, mean (SD)	15.1 ± 2.72	15.57 ± 2.01

### 2.2. Diagnostic criteria

The SUI diagnostic criteria were regarding the recommendations provided by the International Consultation on Urological Diseases for female urinary incontinence (4th edition, 2009):

1. Symptoms: Urine involuntarily leaks out when abdominal pressure increases to various degrees such as laughter, cough, sneezing, or walking, and urine flow terminates immediately when pressurization action is stopped.
2. Signs: When abdominal pressure is increased, urine can be observed to leak out of the urethra involuntarily (pressure-induced test) or positive in 1 hour pad-test, weight gain >1 g.
3. No frequent urination and urgent urination are accompanied by symptoms.

### 2.3. Inclusion criteria

1. Women aged 20 to 60 years old;
2. Meet the above diagnostic criteria;
3. Voluntarily participate in this study and sign an informed consent form;
4. No cognitive impairment or mental illness.

### 2.4. Exclusion criteria

1. Complicated with vaginal or pelvic infection;
2. Severe pelvic pain and vaginal bleeding;
3. Patients with cardiac pacemakers;
4. Patients with pelvic organ prolapse ≥2;
5. Patients suffering from urinary system diseases, such as tumors, stones, and infections;
6. Severe cardiopulmonary dysfunction.

Those who had any of the exclusion criteria will not be included in the study.

### 2.5. Study design and interventions

Before the procedures, all subjects received health education on the etiology and prevention of pelvic floor dysfunction, including prevention and treatment of UI-related knowledge and propaganda and education on a healthy lifestyle. All the patients in the group received basic treatment of pelvic floor biofeedback electrical stimulation during the experiment, each treatment for about 20 to 30 minutes, 6 times a week for 6 weeks. SOKO 900 III therapeutic apparatus (Hailongma Medical Instruments, Beijing, China) was used for pelvic floor rehabilitation, and the biofeedback mode was selected: a vaginal probe was placed into the vagina of the patient, and the muscle fiber comprehensive training (primary) procedure was selected, so as to make the patient actively contract pelvic floor muscles under the guidance of relevant procedures of the therapeutic apparatus. According to the vitality value of pelvic floor muscles after each training, decide whether to enter the next level procedure (the next level will be carried out if the vitality value exceeds 80). After the biofeedback therapy was completed, pelvic floor tissue electrical stimulation therapy was carried out as follows: the electrode piece is attached to the opening beside the midpoint of the line connecting the vaginal introitus and anus of the patient, 1 piece to the left and right. The treatment mode is “SUI,” and the stimulation intensity depends on the tolerance of the patient, which is lower than the degree of stabbing pain.

The patients in the control group were guided by the same experienced rehabilitation therapist to perform the Kegel exercise for 40 minutes each time, 6 times a week for 6 consecutive weeks.

The Kegel exercise: Patients tighten the muscles around the anus, vagina, and urethra. The muscles of the abdomen, buttocks, and thighs are not involved in the contraction as much as possible. It can be performed in any position, and each contraction is 5 seconds, and the relaxation is 5 seconds. Inhale as you contract the muscles and exhale as you relax.

**Figure 2.**
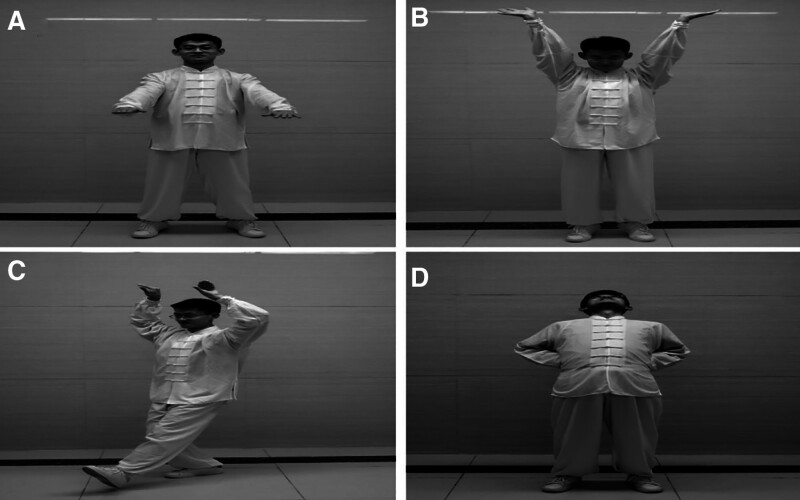
Four selected movements of Mawangdui Guidance. (A) Qishi, (B) Longdeng, (C) Chishi, and (D) Yinyao.

Mawangdui Guidance was carried out in the experimental group. The patients were taught to do Mawangdui Guidance for a total of 40 minutes once a time, including 4 selected movements: “Qishi” (Fig. 2A, raise hands in front of the body), “Longdeng” (Fig. 2B, raise your hands by the side, lift your feet, look down), “Chishi” (Fig. 2C, sit back, stretch your arms up, pull your shoulders back, and lean your head forward), and “Yinyao” (Fig. 2D, body leaning back, head looking up), 6 times per week for 6 consecutive weeks.

All subjects were asked to fill in a daily activity table once a week after the intervention, recording the daily treatment during this week, including drugs, rehabilitation training, and so on to exclude the effect of these treatments during this period.

Calculation of the treatment rate: (actual number/planned total number) × 100%. (If 2/3 of the treatment course has not been completed, will it be regarded as falling off after being agreed by other researchers.)

### 2.6. Evaluation methods

#### 2.6.1. One hour pad-test^[15]^

Before the test, a clean urinal pad with uniform specification was purchased, which was weighed with an electronic scale and recorded the original weight. At the beginning of the test, the bladder of the patient should be filled, and the whole test process should last for 1 hour. The patient should be instructed to stop urination after the test was started. The patient was asked to wear a clean urinal pad or sanitary napkin with the original weight recorded in advance, and instructed the patient to drink 500 mL of water within the first 15 minutes of the whole test; the patient was asked to continue to complete walking and stepping up and down steps within the following 30 minutes; in the last 15 minutes, the patient was instructed to complete 5 tasks, including sitting down-standing up for 10 times, coughing hard for 10 times, running in situ for 1 minute, picking up ground objects for 5 times, and washing hands with tap water for 1 minute. After the test, asked the patient to take off the urinal pad and weigh it, and let the patient urinate and measure the urination volume. The standards for evaluation are as follows:

1. Positive: quantity of fluid loss ≥ 2 g.
2. Mild: 2 g ≤ quantity of fluid loss < 5 g.
3. Moderate: 5 g ≤ quantity of fluid loss < 10 g.
4. Severe: 10 g ≤ quantity of fluid loss < 50 g.
5. Extremely severe: quantity of fluid loss ≥ 50 g.

#### 2.6.2. Pelvic floor muscle function assessment

SOKO 900 III therapeutic apparatus (Hailongma Medical Instruments) was used to evaluate the pelvic floor function of patients. The therapist put a signal acquisition probe into the patient’s vagina, instructed the patient to contract the pelvic floor muscles, and recorded the muscle strength, fatigue, and dynamic pressure of type I and type II muscle fibers.

#### 2.6.3. Incontinence Quality of Life Questionnaire (I-QOL)^[16]^ and International Consultation on Incontinence Questionnaire Urinary Incontinence Short Form (ICI-Q-SF)^[17]^

Patients were asked to recall the leakage of urine in the past 4 weeks and then filled in the questionnaire forms. I-QOL has a total of 22 items, and the scoring standard for each item is 1 to 5 points. The higher the score, the less affected by UI, and the higher the quality of life. ICI-Q-SF was involved in 4 aspects of urine leakage with a total of 21 points. The higher the score is, the more severe the UI is for patients.

#### 2.6.4. Outcome measures^[18]^

1. Obvious effect: the patient did not suffer from urine leakage subjectively or objectively.
2. Effective: quantity of fluid loss in 1 hour pad-test decreased by more than 50% compared with that before.
3. Ineffective: Quantity of fluid loss in 1 hour pad-test decreased by <50% compared with that before.
4. Total effective rate = (obvious effect cases + effective cases)/total cases × 100%.

### 2.7. Statistical analysis

SPSS25.0 was applied to process the related data, the *X*^2^ test was used to compare the counting data, while the measurement data were expressed by mean ± standard deviation (*x* ± *s*). Continuous normal distribution and homogeneity of variance were conducted by *t* test, of which paired sample *t* test was used for intragroup comparison before and after, while independent sample *t* test was used for intergroup comparison. The rank-sum test is used for nonnormal distribution and/or uneven variance. The significance level was α = 0.05.

## 3. Results

### 3.1. Changes in urine leakage before and after treatment in the 1 hour pad-test

Changes in urine leakage before and after treatment in the 1 hour pad-test among the 2 groups are shown in Table 2. The urine leakage of both 2 groups reduced significantly after treatment (*P* < .05), and the improvement of urine leakage in the experimental group is significantly better than that in the control group (*P* < .05).

**Table 2:** Primary and secondary outcome measures for the 2 groups.

Measure	n	Mean ± standard deviation			
		Control group		Experimental group	
		Before intervention	After intervention	Before intervention	After intervention
Urine leakage	30	4.61 ± 2.67	1.22 ± 0.87	4.52 ± 2.39	0.28 ± 0.62*,†
fatigue degree of type I fibers	30	–0.767 ± 10.7	4.53 ± 7.40	–2.97 ± 9.41	1.93 ± 4.33*,†
Fatigue degree of type II fibers	30	–1.83 ± 12.80	5.93 ± 9.42	–0.27 ± 9.78	5.30 ± 6.51*,†
Vaginal dynamic pressure	30	61.37 ± 20.69	96.23 ± 9.88	57.93 ± 12.27	106.57 ± 8.42*,†
I-QOL	30	34.83 ± 6.37	54.33 ± 6.52	35.03 ± 4.97	86.37 ± 7.97*,†
ICI-Q-SF	30	15.1 ± 2.72	5.53 ± 5.71	15.57 ± 2.01	2.93 ± 6.03*,†

* *P* < .05 compared with baseline.

† *P* < .05 experimental group vs the control group.

### 3.2. Changes in pelvic floor muscle fatigue degree before and after treatment

Changes in pelvic floor muscle fatigue degree before and after treatment in the 2 groups are shown in Table 2. The fatigue degree of pelvic floor muscle in the 2 groups is negative before treatment and significantly improved after treatment (*P* < .05). However, in the comparison between the control group and the experimental group, the fatigue degree of pelvic floor muscle type I and type II fibers did not show any difference (*P* > .05). Changes in vaginal dynamic pressure before and after treatment in the 2 groups are shown in Table 2. The pressure in the 2 groups increased significantly after treatment (*P* < .05), and the improvement of vaginal dynamic pressure in the experimental group was significantly better than that in the control group (*P* < .05). Changes in the I-QOL scores and ICI-Q-SF scores before and after treatment in the 2 groups are shown in Table 2. The I-QOL scores of the 2 groups are significantly increased after treatment (*P* < .05), and the growth in the experimental group is significantly higher than that in the control group (*P* < .05). ICI-Q-SF scores among both 2 groups were significantly reduced (*P* < .05), and there was no difference between the 2 groups (*P* > .05).

### 3.3. Before and after treatment in the pelvic floor muscle function test

The pelvic floor muscle strength of the 2 groups before and after treatment in the pelvic floor muscle function test is shown in Tables 3 and 4. The muscle strength of type I and type II pelvic floor muscle fibers of both 2 groups increased after treatment, and the experimental group was better than the control group (*P* < .05). Meanwhile, the number of additional cases with Grade IV and Grade V muscle strength after treatment in the control group was 0 (0%) of type I and 1 (3.33%) of type II, while in the experimental group, 13 (43.33%) of type I and 10 (33.33%) of type II.

**Table 3:** The pelvic floor muscle strength of the 2 groups before and after treatment (n, %).

Group	n	Type	Before						After					
			0	I	II	III	IV	V	0	I	II	III	IV	V
Control group	30	I	4	7	3	2	6	8	0	2	6	8	5	9
		II	4	1	0	3	10	12	0	1	3	3	8	15
Experimental group	30	I	4	12	3	3	4	4	0	0	2	3	6	19
		II	2	1	4	6	11	6	0	1	0	2	3	24
*x* ^2I^			3.649						9.935					
*P* ^I^			.662						.042					
*x* ^2II^			3.249						9.935					
*P* ^II^			.662						.042					

*x*^2I^ and *P*^I^ denote statistical value of type I muscle fibers of pelvic floor muscle; *x*^2II^ and *P*^II^ denote statistical value of type II muscle fibers of pelvic floor muscle.

**Table 4:** The pelvic floor muscle strength of the 2 groups before and after treatment (n, %).

		0	I	II	III	IV	V	*P* value
Control group	I	0	2	6	8	5	9	.042
Experimental group	I	0	0	2	3	6	19	
Control group	II	0	1	3	3	8	15	
Experimental group	II	0	1	0	2	3	24	.042

### 3.4. Comparison of clinical total effective rate

The comparison of clinical efficacy of the 2 groups after treatment is shown in Table 5. The total effective rate of the control group is 76.67%, while the experimental group is 90.00% (*P* < .05).

**Table 5:** The comparison of clinical efficacy of the 2 groups after treatment.

Group	n	Ineffective	Effective	Obvious effect	Total effective rate (%)
Control group	30	7	16	7	76.67
Experimental group	30	3	11	16	90.00
*x* ^2^	6.048				
*P*	.049				

## 4. Discussion

The randomized clinical trial provides evidence that a 6-week Mawangdui Guidance training was effective in improving the function of pelvic floor muscle and vaginal dynamic pressure, meanwhile, symptoms such as urine leakage as well as the quality of life in females with SUI have changed a lot after treatment. However, given that there was no significant difference in fatigue degree of type II fibers of pelvic floor muscle and ICI-Q-SF score between the 2 groups before and after treatment, the reason we concluded was the treatment duration of the study may not be long enough. The results of our study preliminarily demonstrate that biofeedback electrical stimulation of pelvic floor muscle combined with Mawangdui Guidance training has a prospecting value in the clinical treatment of SUI in females.

Risk factors for female SUI include age,^[19,20]^ delivery,^[21]^ pregnancy,^[22]^ obesity,^[23–26]^ chronic cough, and so on. Structural or functional abnormality of the tissue at the base of the pelvis is among the main pathological characteristics of SUI. The nerves and muscle groups, as well as connective tissue of the pelvic floor together, constitute the pelvic support system, which maintains the normal physiological function of the pelvic floor such as keeping the physiological position of pelvic organs and controlling the discharge of defecation and urination. Thus, it is very important for patients to reduce abdominal pressure and strengthen pelvic floor muscles, especially active contraction training of levator anus muscle in the prevention and treatment of SUI. Based on our preexperiment, we assume that maintaining the function of the pelvic support system and improving the physiological structure of the pelvic floor might be the main mechanism for Mawangdui Guidance training to effectively control urine and thus good for the treatment of SUI.

Mawangdui Guidance is an important part of Qigong exercise in traditional Chinese medicine. The 12 movements of Mawangdui Guidance correspond to 12 meridians of traditional Chinese medicine, respectively, and the training of Mawangdui Guidance can not only stimulate acupoints in related meridian but also harmonize the Qi and Blood of the human body. This is the reason why the theory of traditional Chinese medicine holds that Mawangdui Guidance training can strengthen physical fitness and promote the recovery of SUI. Considering the compliance and tolerance of Mawangdui Guidance training for females with SUI, we selected 4 main movements that can treat SUI effectively in Mawangdui Guidance: “Qishi,” “Longdeng,” “Chishi,” and “Yinyao.” “Qishi” is the first movement of Mawangdui Daoyinshu, which mainly includes tucking in the abdomen and buttocks. “Tucking in the abdomen” can effectively activate abdominal muscle groups including superficial rectus abdominis and deep transverse abdominis, what’s more, let them contract together. Studies have shown that this combined contraction method can more effectively improve the pelvic floor muscles’ strength and the function of the pelvic floor, relieve the symptoms of urinary leakage.^[27]^ Also, “tucking in the buttocks” can activate gluteal muscles, pelvic floor muscles, and ligaments. Active contraction training for these muscles can effectively improve the muscle strength and tension of pelvic floor muscles, especially the active contraction of the levator anus, anus, and external urethral sphincter can improve the ability of urine control,^[28–31]^ which is the key strategy of the treatment of SUI. “Longdeng,” the fourth movement of Mawangdui Guidance, is the corresponding Spleen Meridian of Foot-Taiyin. The posture of “Longdeng” consists of a forward body, knee flexion, and squatting, thereby the training of “Longdeng” can improve the urine control ability of levator anus muscle and external urethral sphincter while increasing abdominal pressure. “Chishi,” the seventh movement of Mawangdui Guidance, is the corresponding Bladder Meridian of Foot-Taiyang, and the training of “Chishi” can improve the function of the bladder to discharge urine according to traditional Chinese medicine theory. The key point of “Chishi” is single-leg standing for balance while training, so the ability to control trunk core musculature including the muscles of the waist, abdomen, back, and pelvis floor is of vital importance in this action. Also, the strength of trunk core musculature, pelvic floor muscle, in particular, will be improved gradually. It has been reported that core musculature exercises can enhance the muscle strength of pelvic floor muscle in patients with SUI, and effectively relieve the symptoms of UI and improve the quality of life.^[32,33]^ “Yinyao,” the eighth movement of Mawangdui Guidance, is the corresponding Kidney Meridian of Foot-Shaoyin, and the training of “Yinyao” may be beneficial for kidney health in the sight of traditional Chinese medicine theory. A posture of the forward body, trunk lateral flexion, and twisting of the torso is needed in the training of “Yinyao.” These movements can effectively stretch the lumbar dorsal fascia and exercise the lumbar dorsal muscles, what’s more, providing stability to the spine and joints.^[34]^ It is not hard to understand that this action is beneficial to the stability of pelvic facet joints and enhances the muscle strength of pelvic floor muscles. In a word, the selected movements of Mawangdui Guidance can effectively improve the function of the pelvic support system, enhance the muscle strength and endurance of pelvic floor muscles in females, and then reduce the risk of urine leakage as well as the negative effects on daily life caused by SUI.

However, there are several limitations inherent to our study, such as short observation time, small sample size, various kinds of uncontrollable factors, and so on. A further limitation was the lack of clinic outcomes measures, for instance, a psychological questionnaire on anxiety and depression. In spite of these limitations, to the best of our knowledge, this current study is the first attempt to explore the effects of traditional Chinese medicine Health Qigong Mawangdui Guidance combined with modern rehabilitation therapy to treat SUI in females. Based on our preliminary results, Mawangdui Guidance shows promising effects on the recovery of SUI. Further investigations and researches should be carried out to provide both creditable and effective evidence-based suggestions about Mawangdui Guidance.

## 5. Conclusion

In conclusion, the present study shows that 6-week Mawangdui Guidance exercise had beneficial effects on pelvic floor muscle function and quality of life in females with SUI. Improvements were also observed in the experimental group. Furthermore, Mawangdui Guidance contains 14 sections, we only select 4 movements to analyze. We can make more in-depth research in Mawangdui Guidance. Additionally, many other traditional guidances, such as Taichi, Baduanjin, etc are worthy of applying in the cure to different diseases. And researchers can focus more on these.

## Author contributions

**Data curation:** Zhuan Long.

**Formal analysis:** Yue Zhuo, Kun Ai, Ya Li.

**Funding acquisition:** Ming Xu, Kun Ai.

**Investigation:** Ming Xu, Wanrong Wu.

**Project administration:** Ming Xu.

**Resources:** Shuang Qin.

**Software:** Qiong Liu.

**Supervision:** Ming Xu, Kun Ai, Hong Zhang.

**Writing – original draft:** Ming Xu, Xu Zhang.

**Writing – review & editing:** Ming Xu, Xu Zhang, Hong Zhang.
